# Vascular access for renal replacement therapy among 459 critically ill patients: a pragmatic analysis of the randomized AKIKI trial

**DOI:** 10.1186/s13613-021-00843-3

**Published:** 2021-04-08

**Authors:** Nicolas Benichou, Saïd Lebbah, David Hajage, Laurent Martin-Lefèvre, Bertrand Pons, Eric Boulet, Alexandre Boyer, Guillaume Chevrel, Nicolas Lerolle, Dorothée Carpentier, Nicolas de Prost, Alexandre Lautrette, Anne Bretagnol, Julien Mayaux, Saad Nseir, Bruno Megarbane, Marina Thirion, Jean-Marie Forel, Julien Maizel, Hodane Yonis, Philippe Markowicz, Guillaume Thiery, Frederique Schortgen, Florence Tubach, Jean-Damien Ricard, Didier Dreyfuss, Stéphane Gaudry

**Affiliations:** 1 AP-HP, Hôpital Européen Georges Pompidou, Service de Néphrologie, 75015 Paris, France; 2 Département de Biostatistiques, Santé Publique Et Information Médicale, AP-HP, Hôpital Pitié-Salpêtrière, 75013 Paris, France; 3 INSERM, ECEVE, U1123, CIC 1421, F-75013 Paris, France; 4 Faculté de Médecine Sorbonne, Sorbonne Université, Université, Paris, France; 5 Réanimation Médico-Chirurgicale, CHG, La Roche-sur-Yon, France; 6 Service de Réanimation, CHU de Pointe À Pitre-Abymes, CHU de La Guadeloupe, Pointe-à-Pitre, France; 7 Réanimation Polyvalente, CH René Dubos, 95301 Pontoise, France; 8 Réanimation Médicale CHU Bordeaux, Hôpital Pellegrin, 33000 Bordeaux, France; 9 Service de Réanimation, Centre Hospitalier Sud Francilien, Corbeil Essonne, France; 10 Département de Réanimation Médicale Et Médecine Hyperbare, CHU Angers, Université D’Angers, Angers, France; 11 Réanimation Médicale, CHU Rouen, 76000 Rouen, France; 12 Assistance Publique-Hôpitaux de Paris, Hôpitaux Universitaires Henri Mondor, DHU A-TVB, Service de Réanimation Médicale, Créteil, France; 13 CARMAS Research Group and UPEC-Université Paris-Est Créteil Val de Marne, Créteil, France; 14 Réanimation Médicale, Hôpital Gabriel Montpied, CHU de Clermont-Ferrand, Clermont-Ferrand, France; 15 Réanimation Médico-Chirurgicale, Hôpital de La Source, Centre Hospitalier Régional D’Orléans, BP 6709, 45067 Orléans Cedex, France; 16 Service de Pneumologie Et Réanimation Médicale, APHP, Groupe Hospitalier Pitié-Salpêtrière, Paris, France; 17 Centre de Réanimation, CHU de Lille, Faculté de Médecine, Université de Lille, 59000 Lille, France; 18 Réanimation Médicale Et Toxicologique, Hôpital Lariboisière, INSERM U1144, Université Paris-Diderot, Paris, France; 19 Réanimation Polyvalente, CH Victor Dupouy, 95107 Argenteuil Cedex, France; 20 Service de Réanimation Des Détresses Respiratoires Aiguës Et Infections Sévères, Hôpital Nord Marseille, 13015 Marseille, France; 21 Service de Réanimation Médicale INSERM U1088, Centre Hospitalier Universitaire de Picardie, Amiens, France; 22 Réanimation Médicale, Hôpital de La Croix Rousse, 69004 Lyon, France; 23 Réanimation, CH Cholet, 49300 Cholet, France; 24 Centre Hospitalier Inter-Communal, Service de Réanimation Polyvalente Adulte, Créteil, France; 25 Univ Pierre Et Marie Curie, Sorbonne Universités, 75013 Paris, France; 26 Univ Paris Diderot, Sorbonne Paris Cité, IAME, UMRS 1137, 75018 Paris, France; 27 AP-HP, Service de Réanimation Médico-Chirurgicale, Hôpital Louis Mourier, 92700 Colombes, France; 28 Université de Paris, Paris, France; 29 French National Institute of Health and Medical Research (INSERM), UMR_S1155, Remodeling and Repair of Renal Tissue, Hôpital Tenon, Sorbonne Université, 75020 Paris, France; 30 AP-HP, Service de Réanimation Médico-Chirurgicale, Hôpital Avicenne, 93008 Bobigny, France; 31 Present Address: Service de Réanimation Médico-Chirurgicale, Hôpital Avicenne, 125 Rue de Stalingrad, 93000 Bobigny, France

**Keywords:** Renal replacement therapy, Acute kidney injury, Vascular access, Catheter, Critical care

## Abstract

**Background:**

Vascular access for renal replacement therapy (RRT) is routine question in the intensive care unit. Randomized trials comparing jugular and femoral sites have shown similar rate of nosocomial events and catheter dysfunction. However, recent prospective observational data on RRT catheters use are scarce. We aimed to assess the site of RRT catheter, the reasons for catheter replacement, and the complications according to site in a large population of critically ill patients with acute kidney injury.

**Patients and methods:**

We performed an ancillary study of the AKIKI study, a pragmatic randomized controlled trial, in which patients with severe acute kidney injury (KDIGO 3 classification) with invasive mechanical ventilation, catecholamine infusion or both were randomly assigned to either an early or a delayed RRT initiation strategy. The present study involved all patients who underwent at least one RRT session. Number of RRT catheters, insertion sites, factors potentially associated with the choice of insertion site, duration of catheter use, reason for catheter replacement, and complications were prospectively collected.

**Results:**

Among the 619 patients included in AKIKI, 462 received RRT and 459 were finally included, with 598 RRT catheters. Femoral site was chosen preferentially (*n* = 319, 53%), followed by jugular (*n* = 256, 43%) and subclavian (*n* = 23, 4%). In multivariate analysis, continuous RRT modality was significantly associated with femoral site (OR = 2.33 (95% CI (1.34–4.07), *p* = 0.003) and higher weight with jugular site [88.9 vs 83.2 kg, OR = 0.99 (95% CI 0.98–1.00), *p* = 0.03]. Investigator site was also significantly associated with the choice of insertion site (*p* = 0.03). Cumulative incidence of catheter replacement did not differ between jugular and femoral site [sHR 0.90 (95% CI 0.64—1.25), *p* = 0.67]. Catheter dysfunction was the main reason for replacement (*n* = 47), followed by suspected infection (*n* = 29) which was actually seldom proven (*n* = 4). No mechanical complication (pneumothorax or hemothorax) occurred.

**Conclusion:**

Femoral site was preferentially used in this prospective study of RRT catheters in 31 French intensive care units. The choice of insertion site depended on investigating center habits, weight, RRT modality. A high incidence of catheter infection suspicion led to undue replacement.

**Supplementary Information:**

The online version contains supplementary material available at 10.1186/s13613-021-00843-3.

## Background

The site of vascular access for acute renal replacement therapy (RRT) is a daily clinical question for intensivists and nephrologists taking care of patients with severe acute kidney injury (AKI).

Placement of temporary catheter at the subclavian site is not recommended because of the risk of vascular thrombosis or stenosis of the subclavian vein, which could hamper potential creation of arteriovenous fistula in these patients at risk of end-stage renal failure [1–8].

The CATHEDIA study, a major randomized controlled trial (RCT) comparing femoral and jugular sites for RRT catheter insertion, has shown similar rate of nosocomial event [catheter-tip colonization and catheter-related bloodstream infections (CRBI)] [9] and catheter dysfunction[10]. However, left jugular site was associated with higher rate of dysfunction; whereas, catheter-tip colonization was higher among patients with body mass index (BMI) > 28.4 when femoral position was preferred.

These results, although in contradiction with some previous observational studies [11, 12] and recommendations [13, 14] that advised against femoral site, have led to consider both femoral and jugular sites acceptable. Recent guidelines recommend equally both sites (femoral and jugular) [6, 15] or favor right jugular site in others [16] given that there was a trend in favor of right jugular compared to femoral site regarding dysfunctions in the trial mentioned above.

Since the publication of this RCT and guidelines, there is a scarce of prospective observational data on the habits of intensivists concerning RRT catheter [17].

We published in 2016 a multicenter randomized controlled trial (AKIKI: Artificial Kidney Initiation in Kidney Injury) [18] on RRT timing initiation in intensive care units (ICU) patients with severe AKI (stage 3 of KDIGO classification). The AKIKI trial database provides several interesting prospective data regarding RRT catheters in 31 French ICUs.

We aimed to investigate RRT catheter site, duration of use, reason for catheter replacement, and complications, in particular infectious according to insertion site, in a large population of critically ill patients.

## Methods

### Study design and patients

We performed an ancillary study of the AKIKI trial, an open pragmatic RCT conducted in 31 ICUs in France from September 2013 through January 2016. Adults patients with severe AKI (stage 3 of KDIGO classification) and requirement for invasive mechanical ventilation and/or catecholamine infusion were randomly assigned (1:1) to either an early or a delayed RRT initiation strategy provided they had no life-threatening complication mandating immediate RRT. Detailed protocol is available elsewhere [18]. In the early strategy group, RRT was initiated as soon as possible after randomization. In the delayed group, RRT was initiated only in the context of severe metabolic or clinical abnormalities.

All patients who underwent at least one RRT session were enrolled in the present study.

The original trial was approved by the ethical committee of the French Society of Intensive Care Medicine and by the competent French legal authority (Comité de Protection des Personnes d’Ile de France VI, ID RCB 2013-A00765-40, NCT01932190) for all participating centers. Patients or their surrogates were informed both verbally and with a written document by the investigators. They could refuse to participate at any time, and their decision was recorded in patient files.

### Catheter management

As a pragmatic study, choice of catheter insertion site and management were left at the discretion of each study center and investigator. However, investigators were encouraged to follow the current national guidelines [6], i.e., equivalence of right internal jugular and femoral site in terms of risk for dysfunction or infection, preference for internal jugular site for patients with a body mass index above 28 kg/m^2^ to reduce infectious risk. Guidelines also recommend using catheters of diameter > 12 F and length ≥ 24 cm for femoral site and ultrasound guidance for jugular and femoral vein catheter placement. Removal of RRT catheter was mandated as soon as it is no longer necessary. Information on catheter tip culture at removal, skin cleaning protocols and RRT catheter lock solutions used in participating centers are provided in the Additional file 1: Table S1.

### Endpoints

We sought to describe the sites of RRT catheter insertion, and factors potentially involved in the choice between femoral and jugular for the first catheter (age, weight, sex, SAPS3 and SOFA, acute respiratory distress syndrome (ARDS), invasive mechanical ventilation, hemorrhagic risk (defined as ≥ one of the following criteria: platelets < 100 G/L, prothrombin ratio < 50%, anticoagulation therapy), peripheral vascular disease, RRT modality, investigator site).

We also assessed duration of the first catheter use and reason for replacement (catheter dysfunction, thrombosis, suspicion of infection, proven infection or others, as defined as requiring catheter replacement according to the investigator) according to the insertion site.

Finally, we analyzed complications related to all RRT catheters: pneumothorax requiring exsufflation or drainage, hemothorax, gas embolism, bleeding requiring transfusion or hemostatic procedure, arterio-venous fistula, symptomatic deep venous thrombosis (confirmed with Doppler or CT angiography), bacteremia and fungemia. For RRT catheter associated bloodstream infections, definitions from IDSA 2009 guidelines were used (see Additional file 2: Table S2 [19]).

All data were prospectively collected during the AKIKI trial.

### Statistical methods

Data were expressed as number (percent) for qualitative variables and mean (SD) for quantitative variables. Between groups, Pearson’s Chi-squared tests or Fisher’s exact tests as appropriate were used to compare qualitative variables and Wilcoxon rank sum tests for quantitative variables. To assess the factors potentially involved in the choice between femoral and jugular for the first catheter, a mixed logistic regression model was used. The fixed effects were: randomization arm, age, sex, weight, SAPS3, ARDS, invasive mechanical ventilation, hemorrhagic risk, peripheral vascular disease and the first RRT modality. The investigator study center variable was introduced as random effect. This center effect was evaluated by estimating the corresponding random effect variance component with its 95% confidence interval [20]. We also estimated the intra-class correlation (ICC) interpreted as the percentage of variance explained by the center effect. Collinearity was assessed using the variance inflation factor (VIF) which was estimated for each fixed predictor. A VIF lower than 1.5 was judged adequate.

Kaplan–Meier survival curves were used to describe duration of catheter’s use. To assess the impact of the choice between femoral and jugular for the first catheter on subsequent catheter replacement requirement, we used Fine and Gray competing risk survival model weighted on inverse probability of treatment weights (IPTW). A multivariable logistic regression model was performed to estimate a propensity score for each patient using all covariates described above. To assess weighting balanced measured covariates between the two groups, we used the weighted standardized mean difference, and we considered an absolute standardized difference less than 0.1 as evidence of balance [21]. In the second step, a Fine and Gray model [22], weighted on IPTW, was fitted to take into account the risk of death. The event of interest was the catheter replacement and the competing event was death. Time was defined as delay between dialysis initiation and catheter replacement or death for patients who had one of these events, or duration of catheter without replacement or complication (censored data). Cumulative incidence and subdistribution hazard ratio (sHR) were estimated with their 95% confidence intervals.

All analyses were performed at a two-sided α level of 5% and conducted with R software version 3.3.3 (R Foundation for Statistical Computing).

## Results

### Patient population

**Fig. 1:**
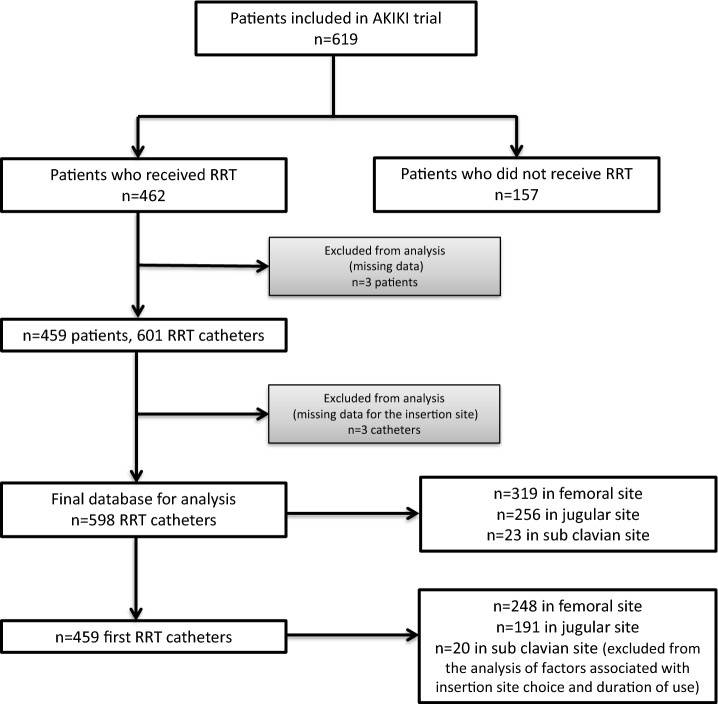
Flow chart

Among the 619 patients included in the AKIKI study, 462 received RRT at least one time. Three were excluded due to missing data, leaving 459 patients in the present study (Fig. 1). A total of 598 catheters were included in the analysis (3 were excluded due to missing data).

Patient characteristics are detailed in Table 1.

**Table 1:** Characteristics of the patients at baseline

Characteristics	Patients (*n* = 459)
Sex (men/women) *n* (%)	310/149 (67.5 / 32.5)
Age (year) (± SD)	65.6 (± 13.7)
Weight (± SD)	85.5 (± 22.2)
Chronic renal failure^a^ *n* (%)	43 (9)
Congestive heart failure *n* (%)	41 (9)
Ischemic heart disease *n* (%)	43 (9)
Hypertension *n* (%)	235 (51)
Diabetes mellitus *n* (%)	121 (26)
Invasive mechanical ventilation *n* (%)	394 (86)
ARDS^b^ *n* (%)	156/456 (34)
SAPS III^c^ (± SD)	73.4 (± 14.0)
SOFA^d^ (± SD)	11.2 (± 3.0)
Central venous catheter *n* (%)	411 (90)

All data at enrollment. Plus–minus values are means ± SD (standard deviation)

^a^Chronic renal failure defined as eGFR < 60 ml/mn

^b^ARDS: Berlin definition

^c^The Simplified Acute Physiology Score (SAPS) III ranges from 0 to 146, with higher scores indicating more severe disease and a higher risk of death

^d^The Sepsis‐related Organ Failure Assessment (SOFA) score ranges from 0 to 24, with higher scores indicating more severe organ failure

### Choice of RRT catheter insertion site

Table 2 shows the number of RRT catheters according to the insertion site. Femoral site was chosen in 53% (*n* = 319) of cases, jugular site in 43% (*n* = 256). As expected, the choice of subclavian site was very rare (less than 5%, *n* = 23). For the first catheter, among the 439 catheters placed in jugular or femoral site (considering that for the subclavian placement, jugular or femoral was not an option), the sample proportion of 248/439 femoral placement (56.5%, 95% CI 51.7–61.2) was statistically significantly different from a 50% expected proportion with *p* = 0.008 (*z* test to compare a single proportion to population estimate).

**Table 2:** Number of catheters according to insertion site

Insertion site	Total	First	Second	Third and ≥
Femoral	319 (53%)	248 (54%)	49 (52%)	22 (43%)
Jugular	256 (43%)	191 (42%)	44 (46%)	21 (53%)
Sub-clavian	23 (4%)	20 (4%)	2 (2%)	1 (3%)
Total	598 (100%)	459	95	44

In multivariate analysis (Table 3), continuous RRT modality was significantly associated with preference of femoral site as the first choice [OR = 2.22 (95% CI 1.28–3.86), *p* = 0.005]. On the opposite, higher weight was associated with preference for jugular site [OR = 0.99 (95% CI 0.98–0.99), *p* = 0.03]. Investigator site was also significantly associated with the choice of insertion site (between femoral and jugular) in multivariate analysis (*p* = 0.03). The percentage of variance explained by the investigator center was of 10.5%.

**Table 3:** Factors potentially associated with the choice of insertion site in univariate and multivariate analyses (between femoral and jugular for the first catheter, *n* = 439)

Variable^f^	Femoral (*n* = 248)	Jugular (*n* = 191)	Univariate analysis	Multivariate analysis	
			*p* values^a^	OR (CI 95)	*p* values^b^
Randomization arm:delayed group	81 (32.7%)	68 (35.6%)	0.52	0.97 (0.59–1.58)	0.89
Age—years	64.51 (14.62)	66.21 (12.54)	0.44		
Age > 70 years	103 (41.5%)	77 (40.3%)	0.80	1.08 (0.66–1.76)	0.78
Sex (Male)	174 (70.2%)	122 (63.9%)	0.16	1.43 (0.89—2.37)	0.16
Weight at randomization—mean (SD)	83.21 (19.9)	88.92 (25.2)	0.07	0.99 (0.98–0.99)	0.03
Peripheral vascular disease	35 (14.1%)	21 (11%)	0.33	1.46 (0.70–3.07)	0.32
ARDS at randomization	88/246 (35.8%)	61/190 (32.1%)	0.42	1.00 (0.59–1.68)	0.99
SAPS 3 score at randomization – mean (SD)	73.6 (15.5)	73.5 (12.5)	0.81	1.00 (0.98 – 1.01)	0.58
Invasive mechanical ventilation	211 (85.1%)	164 (85.9%)	0.82	0.85 (0.41 – 1.73)	0.38
Hemorrhagic risk ^c^	172/225 (76.4%)	124/166 (74.7%)	0.69	1.01 (0.59 – 1.72)	0.98
Modality of first RRT Continuous	125 (50.6%)	75 (39.3%)	0.02	2.22 (1.28 – 3.86)	0.005
Investigator center^d^	–	–	–	–	0.03^e^

^a^*p* values: Pearson's Chi-squared for qualitative data and Wilcoxon rank sum test for quantitative data test.

^b^*p* values: associated to multivariate logistic regression model.

^c^Hemorrhagic risk: ≥ one of the following criteria: platelets < 100 G/L, prothrombin ratio < 50%, anticoagulation therapy.

^d^Investigator center is a variable with 32 levels.

^e^*p* value associated with investigator center: This center effect was evaluated by estimating the corresponding random effect variance component. The variance of center variable was of 0.38 (95% CI, 0.28–0.89). We also estimated the percentage of variance explained by the effect center and it was of 10.5%

^f^Collinearity control: variance inflation factor (VIF) was lower than 1.5, thus judged adequate, for each fixed predictor (Additional file 3: Table S3).

### Duration of use and reasons for catheter replacement

**Fig. 2:**
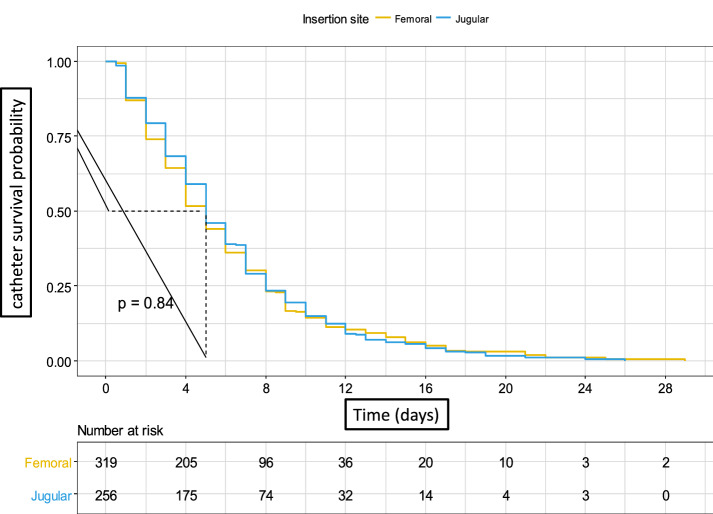
Duration of catheter use. Legend: Median duration of use was 5 days for both femoral (IQR 2–8), and jugular (IQR 3–8) catheters (*p* = 0.84)

Overall median duration of catheter use was 5 days for both femoral (IQR 2–8), and jugular (IQR 3–8) catheters (log rank test *p* = 0.84, Fig. 2).

In a Fine and Gray competing risk model analysis weighted on inverse probability weighting treatment with death as competing event (Additional file 4: Table S4, Additional file 6: Figure S2), probability of first catheter replacement did not differ between femoral and jugular site [sHR = 0.90 (95% CI 0.64–1.25), *p* = 0.67, Additional file 5: Figure S1].

Cumulative incidence of first catheter replacement at day 7 were, respectively, 0.2 (95% CI 0.14–0.26) for femoral and 0.19 (95% CI 0.12–0.25) for jugular site (*p* = 0.77, Table 4).

**Table 4:** Cumulative incidence of catheter replacement and death according to insertion site (first catheter, jugular or femoral)

Event	Site	Cumulative incidence (CI 95)			*p* value
		Day 7	Day 14	Day 21	
Catheter replacement	Femoral	0.2 (0.14–0.26)	0.29 (0.2–0.37)	0.41 (0.29–0.53)	0.770
	Jugular	0.19 (0.12–0.25)	0.34 (0.24–0.45)	0.37 (0.25–0.48)	
Death	Femoral	0.23 (0.17–0.29)	0.36 (0.27–0.45)	0.39 (0.29–0.5)	0.590
	Jugular	0.21 (0.14–0.27)	0.3 (0.21–0.39)	0.35 (0.22–0.48)	

Reasons for first catheter replacement (Table 5) were not different for femoral and jugular sites. The main reason was dysfunction [26/49 for femoral (53.1%), 16/36 for jugular (44.4%)]. All sub-clavian catheter replacements resulted from dysfunction. The absolute rates of replacement for dysfunction among all catheters were 10.4% (26/248) for femoral and 8.4% (16/191) for jugular site.

**Table 5:** Reasons for first catheter replacement according to insertion site

Reason for catheter replacement	Total	Femoral	Jugular	Sub-clavian
Dysfunction	47 (52.2%)	26 (53.1%)	16 (44.4%)	5 (100%)
Thrombosis	1 (1.1%)	1 (2%)	0 (0%)	0 (0%)
Catheter infection suspicion	29 (32.2%)	14 (28.6%)	15 (41.7%)	0 (0%)
Proven catheter infection	4 (4.4%)	1 (2%)	3 (8.3%)	0 (0%)
Other	9 (10%)	7 (14.3%)	2 (5.6%)	0 (0%)
Total	90	49	36	5

Of note, suspected infection led to 14 (28.6%) replacements in femoral group and 15 (41.7%) in jugular group; whereas, infection leading to replacement was documented in only 1 and 3 instances for femoral and jugular sites, respectively.

### Catheter-related complications

Catheter-related complications according to insertion site are detailed in Table 6.

**Table 6:** Possible catheter-related complications according to insertion site (all catheters)

	Femoral (*n* = 319 catheters)	Jugular (*n* = 256 catheters)	Sub-clavian (*n* = 23 catheters)	*p* values (femoral vs jugular)
*Infectious complications*				
Catheter-related bloodstream infections				
Number^a^	6 (1.9%)	7 (2.7%)	0	0.58
Incidence rate (%)^b^	2.2%	3.2%		–
Incidence density (‰ catheter-days)^c^	3.1‰	4.4‰		–
Bloodstream infections without cause^a^	5 (1.6%)	1 (0.4%)	0	0.23
*Mechanical complications*^a^				
Pneumothorax	0	0	0	1.00
Hemothorax	0	0	0	1.00
Gas embolism	1 (0.3%)	0	0	1.00
Fistula	1 (0.3%)	0	0	1.00
Bleeding requiring transfusion or hemostatic procedure^a^	2 (0.6%)	2 (0.8%)	0	1.00
*Thrombotic complications*^a^				
Symptomatic deep venous thrombosis^a^	2 (0.6%)	3 (1.2%)	0	0.66

All complications above are «catheter related» except «bloodstream infections without cause»

*P* values: Fisher’s exact test for count data. No *p* value calculated for incidence rate and incidence density because the two samples were not independent. Patients could have a femoral and jugular catheter

^a^All percentages are expressed as number of events per number of catheters except for incidence rate and incidence density

^b^Incidence rate: number of patients contracting an infection per number of patients at risk (272 patients under femoral catheter and 221 patients under jugular catheter)

^c^Incidence density: number of infection per 1000 catheter-days

Rates of catheter-related bloodstream infections (bacteremia or fongemia) were rare and not different between femoral and jugular sites (respectively, 3.1‰ and 4.4‰ catheter-days). Rates of catheter-related infections according to randomization arm (*i.e., *RRT initiation strategy) are provided in the Additional file 7: Table S7.

No pneumothorax nor hemothorax directly related to RRT catheter insertion occurred during the study. One catheter-related gas embolism and 1 catheter-related fistula occurred in the femoral site. Rate of catheter-related bleeding requiring transfusion or hemostatic procedure was very rare and similar in both sites [2 for femoral (0.6%) and 2 for jugular (0.8%)]. Finally, we observed very few symptomatic deep venous thromboses (2 among femoral catheters, and 3 among jugular ones).

## Discussion

In this prospective study of RRT catheters among critically ill patients with severe acute kidney injury in 31 French ICUs, femoral site was preferentially used, before jugular site. The choice of insertion site depended on RRT modality (femoral site was more frequently chosen for continuous modality), patient weight (jugular site was preferred for higher weight patients) and investigator study center habits. The rate of dysfunction and complications did not differ between jugular and femoral catheters. Suspicion of infection led to replacement of many catheters but was actually seldom proven, and the incidence of clinically significant infection was quite low.

The main objective of this study was to show a «real-life» use of RRT catheters in a large population of ICU patients. We took advantage of our recently published large multicenter randomized controlled trial (AKIKI) involving severely ill patients (acute kidney injury stage 3 and mostly receiving mechanical ventilation and catecholamine infusion), although one should note that patients with life-threatening complications of severe AKI such as severe hyperkalemia were excluded from the trial, thus limiting generalizability of our findings in this population. Few years after the publication of an important RCT on the topic of RRT catheters [9, 10], the present study provides an interesting snapshot of RRT catheter use in the ICU in France. Furthermore, one should note that our study population included more severe patients than the aforementioned trial (85% receiving catecholamines *vs* 35–40%), thus providing interesting data in this particular population.

The small difference regarding the choice of the insertion site (between femoral and jugular) is probably the consequence of the guidelines [6, 15] leaving the choice between the 2 sites. These guidelines are based on the results of the CATHEDIA study [9, 10, 23]. This French multicenter RCT published in 2008 included 736 dialyzed ICU patients and found no difference in terms of infectious complications (colonizations and bloodstream infections) [9] nor in dysfunctions of catheters [10] between these two sites. Choice of the site of insertion for the second catheters, which were probably placed in less urgent situations, were similar. These findings suggest that the conclusions of this trial have been adopted by clinicians, who do not hesitate to use femoral site.

Of note, subclavian site was the choice for 4% of the first catheters used. This site has been prohibited in every nephrology recommendation for many years, because of the important risk of thrombosis or stenosis of the subclavian vein. Indeed, such a complication hampers a potential creation of arteriovenous fistula, a major issue in patients with AKI who are now considered to be at risk of end stage renal failure [1–8].

Higher weight was associated with preference for jugular site insertion, in agreement with the demonstration of a significantly lower incidence of colonization in the jugular compared to femoral site by the CATHEDIA study sub-group analysis [9] in patients with a BMI > 28.4. Noteworthy, however, is the fact that we did not record BMI. To our knowledge, our study is the first to show that RRT modality impacts the choice of RRT catheter site, continuous RRT (CRRT) being associated with more femoral catheterization. This finding fits with the higher proportion of femoral RRT catheters (67%) used in the RENAL study [24] compared to our trial, as it assessed optimal dialysis dose among CRRT-treated patients only. One should hypothesize that patients receiving intermittent RRT modality are more susceptible to be mobilized (sitting position) between the sessions. In this condition, some intensivists may avoid femoral site due to the risk of femoral thrombosis.

Quite surprisingly, hemorrhagic risk (defined as at least one of the following criteria: platelets < 100 G/L, prothrombin ratio < 50%, curative anticoagulation therapy) was not associated with insertion site, unlike results from a secondary analysis of the ATN trial [25], that showed a tendency to place more femoral catheters in coagulopathic patients. It should be mentioned that the use of antiplatelets agents was not recorded in our study though.

We acknowledge that central venous infusion catheters site (femoral or jugular) was not known at randomization in the AKIKI trial. This could be an important factor involved in the choice of RRT catheter insertion site, given that 90% of patients had central venous lines at baseline.

The first reason for first catheter replacement was dysfunction (approximately half of cases) in both jugular and femoral site. Rate of dysfunction leading to catheter replacement among first catheters (around 10%) was similar to that previously reported in the literature, notably in the CATHEDIA study [10]. Unfortunately, we were not able to provide the information of left versus right jugular insertion. One should know that in the CATHEDIA trial, dysfunctions were more frequent in left jugular site than in right jugular and femoral sites.

We found a high incidence (around 30%) of catheter replacement related to infection suspicions; whereas, only a few infections were actually proven (around 4% of catheter replacements). This underlines the lack of reliable diagnostic technique for catheter-related infections [26, 27]. These unnecessary changes of catheters have negative consequences such as waste of time and inherent risks of new catheter insertion.

Only 6 (3.1‰ catheter-days) catheter-related bloodstream infections in femoral and 7 (4.4‰ catheter-days) in jugular site were found. These are rather low rates (even if we aggregate the number of catheter-related bloodstream infections with those of unknown origin) in this population of very severe and fragile patients, especially given the fact that RRT catheters might be associated with more CRBIs than other central catheters [28]. These results, similar to those from recent studies [29, 30], reflect the progress achieved during the last years regarding infection prevention and catheter care [31, 32], due to generalization of infection-control «bundles», mainly sterile precautions at the time of catheter insertion and at each manipulation [6, 16, 33–36]. The short duration of catheter use in our study (median 5 days) also highlights the importance of prompt removal of unnecessary catheters. Even more importantly, it seems obvious that the best way to prevent catheter-related infection is to avoid unnecessary catheter insertion. This is particularly true in that study which stems from an RCT showing the inutility of 50% of RRT initiation: indeed, there were twice as many patients with catheter-related bloodstream infection in the early initiation arm (10%) compared to the delayed strategy (5%) of the AKIKI trial [18].

No mechanical complication (pneumothorax and hemothorax) related to RRT catheters occurred, among our 459 patients, 598 catheters (including 256 jugular and 20 subclavian insertion sites). This may be due to the generalization of ultrasound guidance for catheter placement [37] which was strongly recommended at the time of AKIKI (6, 16, 38–40) for jugular site and for femoral site although we did not record the proportion of ultrasound guidance in the present study.

Our study has potential limitations, mostly driven by its ancillary design, within a RCT not directly aiming at investigating RRT catheters. First, proportions of left and right jugular catheters were not collected. Second, use of echography to guide catheter insertion was not recorded. Third, position (femoral or jugular) of central venous catheters already in place for fluid and drug administration at randomization was not known. This could have influenced the choice of RRT catheter insertion site. Fourth, the definitions of catheter dysfunction, thrombosis and suspicion of infection were not prespecified during the AKIKI trial but left to the appreciation of investigators. Fifth, duration or intervals between RRT sessions and clinical surveillance of insertion sites were left at discretion of the physicians, which might have influenced rate and time to catheter replacement. Of note, analyses regarding exposures associated with insertion site, rate and time to catheter replacement were limited to first catheter per patient, limiting generalizability of our findings to this situation.

## Conclusion

Femoral site was preferentially used in this observational study of RRT catheters in 31 French intensive care units. The choice of insertion site depended on RRT modality, patient weight and investigating center habits. There were similar rates of dysfunctions and complications between femoral and jugular sites.

We found a high incidence of undue catheter replacement related to infection suspicion, whereas only a few proven catheter infections actually occurred. Therefore, one interesting research focus could be trying to develop more accurate diagnosis techniques of catheter related infections to avoid undue RRT catheter changes. Unnecessary RRT should be avoided to minimize catheter-related complications.

## Supplementary Information

**Additional file 1: Table S1.** Catheter tip culture at removal, skin cleaning protocol and catheter lock solution according to participating centers.

**Additional file 2: Table S2.** Catheter-related bacteremia or fungemia definitions (from Guidelines IDSA 2009, CID 2009 Dec 1 ;49:1-45).

**Additional file 3: Table S3.** Collinearity control: variance inflation factors for fixed effects factors potentially involved in the choice between femoral and jugular for the first catheter insertion.

**Additional file 4: Table S4.** Covariate balance between femoral and jugular groups before and after propensity score weighting for first catheter insertion.

**Additional file 5: Figure S1.** Cumulative incidence of catheter replacement and death, for first catheter, according to insertion site (jugular and femoral).

**Additional file 6: Figure S2.** Propensity score distribution between femoral and jugular groups for first catheter insertion.

**Additional file 7: Table S5.** Possible infectious catheter-related complications according to insertion site and arm of randomization (among patients who underwent RRT and catheter insertion).

## Data Availability

The datasets used during the current study are available from the corresponding author on reasonable request.

## Abbreviations

RRT: Renal Replacement Therapy
AKI: Acute Kidney Injury
RCT: Randomized Controlled Trial
ICU: Intensive Care Unit
AKIKI: Artificial Kidney Initiation in Kidney Injury
CRBI: Catheter Related Bloodstream Infections
BMI: Body Mass Index
KDIGO: Kidney Disease Improving Global Outcomes
SAPS: Simplified Acute Physiology Score
SOFA: Sepsis‐related Organ Failure Assessment
ARDS: Acute Respiratory Distress Syndrome
