# Supplementary material for: Vascular access for renal replacement therapy among 459 critically ill patients: a pragmatic analysis of the randomized AKIKI trial
Source: Ann Intensive Care. 2021 Apr 8;11:56. doi: 10.1186/s13613-021-00843-3 (PMC8032839; doi:10.1186/s13613-021-00843-3)
Supplement: Supplementary file 1 — Additional file 1: Table S1. Catheter tip culture at removal, skin cleaning protocol and catheter lock solution according to participating centers. [file 13613_2021_843_MOESM1_ESM.docx]

# Additional file 1

Table S1. Catheter tip culture at removal, skin cleaning protocol and catheter lock solution according to participating centers

|  | Number of study ~~site~~ center  (among the 17 sites which included more that 10 patients) | Percentage of study ~~site~~ center |
| --- | --- | --- |
| Systematic catheter tip culture at removal | 9/17 | 53% |
| Skin cleaning protocol |  |  |
| Chlorhexidine | 9/17 | 53% |
| Alcoholic povidone iodine | 8/17 | 47% |
| Catheter lock solution |  |  |
| 0.9% NaCl | 6/17 | 35% |
| Citrate | 4/17 | 24% |
| Heparine | 5/17 | 29% |
| Taurolidine - citrate | 1/17 | 6% |
| Water for injection | 1/17 | 6% |

Data not available for 2 additional participating centers
