# Supplementary material for: Vascular access for renal replacement therapy among 459 critically ill patients: a pragmatic analysis of the randomized AKIKI trial
Source: Ann Intensive Care. 2021 Apr 8;11:56. doi: 10.1186/s13613-021-00843-3 (PMC8032839; doi:10.1186/s13613-021-00843-3)
Supplement: Supplementary file 2 — Additional file 2: Table S2. Catheter-related bacteremia or fungemia definitions (from Guidelines IDSA 2009, CID 2009 Dec 1 ;49:1-45). [file 13613_2021_843_MOESM2_ESM.docx]

# Additional file 2

| Table S2. Catheter-related bacteremia or fungemia definitions (from Guidelines IDSA 2009, CID 2009 Dec 1 ;49 :1-45)   |
| --- |
