# Supplementary material for: Vascular access for renal replacement therapy among 459 critically ill patients: a pragmatic analysis of the randomized AKIKI trial
Source: Ann Intensive Care. 2021 Apr 8;11:56. doi: 10.1186/s13613-021-00843-3 (PMC8032839; doi:10.1186/s13613-021-00843-3)
Supplement: Supplementary file 3 — Additional file 3: Table S3. Collinearity control: variance inflation factors for fixed effects factors potentially involved in the choice between femoral and jugular for the first catheter insertion. [file 13613_2021_843_MOESM3_ESM.docx]

# Additional file 3

Table S3. Collinearity control : variance inflation factors for fixed effects factors potentially involved in the choice between femoral and jugular for the first catheter insertion

| Variable | VIF |
| --- | --- |
| Randomization arm | 1.02 |
| Age > 70 years | 1.07 |
| Sex | 1.05 |
| Weight at randomization | 1.05 |
| Peripheral vascular disease | 1.06 |
| ARDS at randomization | 1.06 |
| SAPS 3 score at randomization | 1.11 |
| Invasive mechanical ventilation | 1.07 |
| Hemorrhagic risk | 1.03 |
| Modality of first RRT | 1.06 |

A VIF lower than 1.5 was judged adequate.
