# Supplementary material for: Vascular access for renal replacement therapy among 459 critically ill patients: a pragmatic analysis of the randomized AKIKI trial
Source: Ann Intensive Care. 2021 Apr 8;11:56. doi: 10.1186/s13613-021-00843-3 (PMC8032839; doi:10.1186/s13613-021-00843-3)
Supplement: Supplementary file 4 — Additional file 4: Table S4. Covariate balance between femoral and jugular groups before and after propensity score weighting for first catheter insertion. [file 13613_2021_843_MOESM4_ESM.docx]

# Additional file 4

Table S4. Covariate balance between femoral and jugular groups before and after propensity score weighting for first catheter insertion

| Variable | Standardized mean Differences | |
| --- | --- | --- |
|  | Naive | Weighted |
| Sex | 0.16 | 0.01 |
| Age | 0.05 | 0.01 |
| Weight at randomization | 0.19 | 0.02 |
| Randomization arm | 0.02 | 0.01 |
| Peripheral vascular disease | 0.15 | 0.03 |
| SAPS 3 score at randomization | 0.02 | 0.02 |
| ARDS at randomization | 0.05 | 0.03 |
| Invasive mechanical ventilation | 0.11 | 0.01 |
| Hemorrhagic risk | 0.02 | 0.003 |
| Modality of first RRT | 0.38 | 0.02 |

All standardized mean differences after propensity score weighting < 0.1, i.e. good balance between femoral and jugular groups
