# Supplementary material for: Vascular access for renal replacement therapy among 459 critically ill patients: a pragmatic analysis of the randomized AKIKI trial
Source: Ann Intensive Care. 2021 Apr 8;11:56. doi: 10.1186/s13613-021-00843-3 (PMC8032839; doi:10.1186/s13613-021-00843-3)
Supplement: Supplementary file 5 — Additional file 5: Figure S1. Cumulative incidence of catheter replacement and death, for first catheter, according to insertion site (jugular and femoral). [file 13613_2021_843_MOESM5_ESM.docx]

# Additional file 5

Figure S1. Cumulative incidence of catheter replacement and death, for first catheter, according to insertion site (jugular and femoral)


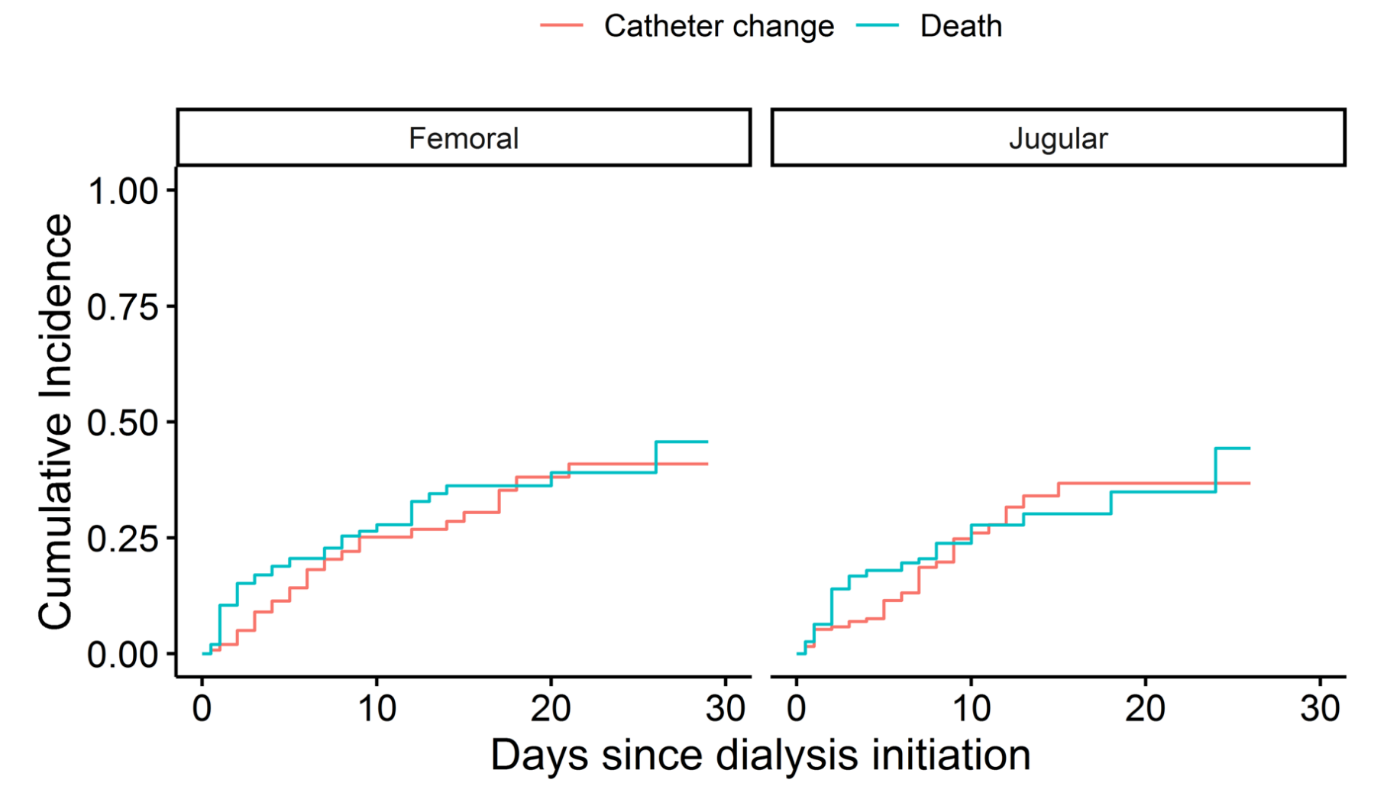


Competing risk survival model : the event of interest was the catheter replacement and the competing event was death. Time was defined as delay between dialysis initiation and catheter replacement or death for patients who had one of these events, or catheter removal without catheter change for the others.

For catheter replacement, sub Hazard Ratio was 0.94 (95% CI 0.62 - 1.43), p = 0.77.

For death, sub Hazard Ratio was 0.89 (95% CI 0.61 - 1.31), p = 0.57.
