# Supplementary material for: Vascular access for renal replacement therapy among 459 critically ill patients: a pragmatic analysis of the randomized AKIKI trial
Source: Ann Intensive Care. 2021 Apr 8;11:56. doi: 10.1186/s13613-021-00843-3 (PMC8032839; doi:10.1186/s13613-021-00843-3)

# Additional file 6

Figure S2. Propensity score distribution between femoral and jugular groups for first catheter insertion


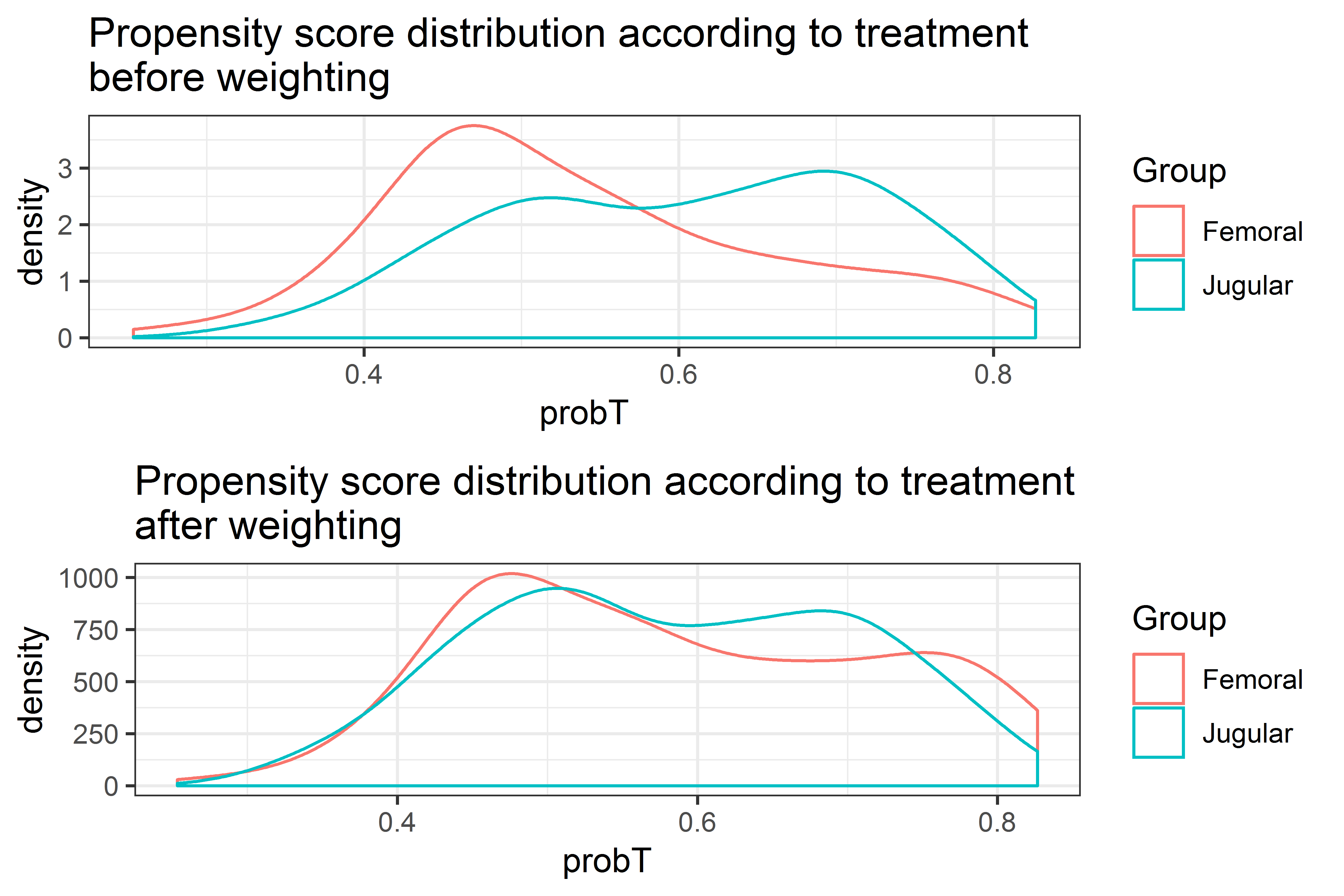

Supplement: Supplementary file 6 — Additional file 6: Figure S2. Propensity score distribution between femoral and jugular groups for first catheter insertion. [file 13613_2021_843_MOESM6_ESM.docx]
