# Supplementary material for: Vascular access for renal replacement therapy among 459 critically ill patients: a pragmatic analysis of the randomized AKIKI trial
Source: Ann Intensive Care. 2021 Apr 8;11:56. doi: 10.1186/s13613-021-00843-3 (PMC8032839; doi:10.1186/s13613-021-00843-3)
Supplement: Supplementary file 7 — Additional file 7: Table S5. Possible infectious catheter-related complications according to insertion site and arm of randomization (among patients who underwent RRT and catheter insertion). [file 13613_2021_843_MOESM7_ESM.docx]

# Additional file 7

Table S5. Possible infectious catheter-related complications according to insertion site and arm of randomization (among patients who underwent RRT and catheter insertion)

|  | **Femoral, early arm (n = 220 catheters)** | **Femoral, delayed arm (n = 99 catheters)** | **Jugular, early arm (n = 165 catheters)** | **Jugular, delayed arm (n = 91 catheters)** |
| --- | --- | --- | --- | --- |
| **Catheter-related bloodstream infections** |  |  |  |  |
| Number^1^ | 5 (2.3%) | 1 (1.0%) | 5 (3.0%) | 2 (2.2%) |
| Incidence rate (%)^2^ | 2.7% | 1.1% | 3.5% | 2.5% |
| Incidence density (‰ catheter-days)^3^ | 3.7‰ | 1.7‰ | 5.0‰ | 3.4‰ |
| **Bloodstream infections without cause^1^** | 2 (0.9%) | 3 (3.0%) | 0 (0.0%) | 1 (1.1%) |

^1^All percentages are expressed as number of events per number of catheters except for incidence rate and incidence density

^2^Incidence rate: number of patients contracting an infection per number of patients at risk

^3^Incidence density : number of infection per 1000 catheter-days
